# Development of EST-SSR primers and genetic diversity analysis of the southern blight pathogen *Sclerotium rolfsii* using transcriptome data

**DOI:** 10.3389/fmicb.2023.1152865

**Published:** 2023-05-30

**Authors:** Fanfan Wang, Tao Tang, Ting Mao, Yuanyuan Duan, Xiaoliang Guo, Jingmao You

**Affiliations:** ^1^Key Laboratory of Biology and Cultivation of Chinese Herbal Medicines, Ministry of Agriculture and Rural Affairs, Institute of Chinese Herbal Medicines, Hubei Academy of Agricultural Sciences, Enshi, China; ^2^Hubei Engineering Research Center of Under-forest Economy, Hubei Academy of Agricultural Sciences, Wuhan, China; ^3^Hubei Engineering Research Center of Good Agricultural Practices (GAP) Production for Chinese Herbal Medicines, Institute of Chinese Herbal Medicines, Hubei Academy of Agricultural Sciences, Enshi, China

**Keywords:** southern blight, *Sclerotium rolfsii*, molecular markers, genetic diversity, transcriptome

## Abstract

**Introduction:**

*Sclerotium rolfsii Sacc*. is a globally dispersed pathogenic fungus that causes southern blight disease in many crops and Chinese herbal medicine. The high degree of variation and diversity in the fungi altered population genetic structure. Therefore, the important factors of variation within the pathogen population should be considered during the development of management strategies for the disease.

**Methods:**

In this study, *S. rolfsii* isolates from 13 hosts in 7 provinces of China were collected and analyzed to identify their morphological features and perform molecular characterization. To develop EST-SSR primers, transcriptome sequencing was performed on isolated CB1, and its SSR loci were comprehensively analyzed. In addition, we analyzed the polymorphisms among different populations based on screened EST-SSR primers.

**Results:**

The results showed that all of these clean reads with total 36,165,475 assembled bases were clustered into 28,158 unigenes, ranged from 201 bp to 16,402 bp on the length, of which the average length was 1,284 bp. Of these, the SSR sequence appeared at an average interval of 15.43 kB, and the frequency of SSR was 0.0648 SSR/kB. Polymorphism of 9 primers was observed among 22 populations, and was verified by the Shannon’s index (average = 1.414) and polymorphic information index (> 0.50). The genetic diversity analysis revealed diversity in all host populations and geographical populations. Further, molecular variance analysis (AMOVA) showed that the differences between groups were mainly related to geographical location. Based on cluster analysis, the 7 populations were roughly divided into 3 groups, and the results were highly consistent with those based on the geographical location, ultimately aligning with the results of STRUCTURE analysis.

**Discussion:**

The findings build on current knowledge of the distribution of *S. rolfsii* in the southwest area of China, adding value to current knowledge base on the population structure and genetic diversity of *S. rolfsii*, specifically in the context of Chinese herbal medicine cultivation in China. Overall, our findings may provide valuable information for breeding of crops with enhanced resistance toward *S. rolfsii*.

## 1. Introduction

The occurrence of southern blight on plants is caused by *Sclerotium rolfsii* Sacc. (Teleomorph: *Athelia rolfsii* (Cruzi) Tu & Kimbrough) worldwide (Dwivedi and Prasad, 2016). The soil-borne fungus can infect crop, vegetables, fruits, Chinese medicinal plant, and other 500 types of plant species, and was first described by Rolfs in 1892 on tomato (Dwivedi and Prasad, 2016). Southern blight is very severe and among one of the main significant diseases worldwide in terms of economic losses in temperate regions with cool and moist climate during spring. The yield loss of in peanut production owing to *S. rolfsii* infection was found to range from 10 to 80% all over the world (Khatri et al., 2017; Song et al., 2018). The initial symptoms in plant were brown to black rot of the root or stem and colonization of the lesions by the fast-growing mycelium. Once the disease continues to progress, the infected strain dies and its mycelium spreads in all directions (Mehri et al., 2020). The pathogen overwinters as sclerotia in soil, and as mycelium in seedlings and plant survival (Ünal et al., 2019). In early spring, when temperature and moisture are suitable, the sclerotium germinates to produce mycelium, which is spread through rainwater, irrigation water, etc., directly invading plants and causing disease (Li et al., 2021).

The disease is difficult to handle due to its extensive presence, high variation, and ability to produce persistent sclerotia (Remesal et al., 2012; Xie et al., 2014; Jebaraj et al., 2017). During the development of management strategies for the disease, variation within the pathogen population, which is an important factor, should be considered (Khatri et al., 2017). Currently, the control measures for this disease mainly depend on chemical fungicides, which not only lead to fungicide resistance, but also pollute the environment (Sultana and Hossain, 2022). An improved understanding of *S. rolfsii* populations in plants could provide more information on approaches to manage this devastating pathogen while improving control efficiency and reducing the use of fungicides. Therefore, the genetic diversity of the pathogen, and their correlation with host and geographical distribution are research hotspots.

Previous studies revealed the variability among the isolates of *S. rolsfii* from different hosts and geographical locations in terms of phenotypic traits (Sarma et al., 2002; Remesal et al., 2012; Xie et al., 2014). However, the methods used to obtain these results are laborious, time-consuming, and subject to environmental influences; hence, they are not generally preferred. Molecular markers are more effective for evaluating the genetic diversity of fungal pathogens than conventional methods, and are widely used in plant pathology, such as in the analysis of the origin, migration pathways, and genetic structure of wheat stripe rust, and population genetic analysis of *Sclerotinia sclerotiorum* in Northeast China (Rathour et al., 2004; Luo et al., 2015; Liu et al., 2018). Currently used molecular markers include restriction fragment length polymorphism (RFLP), random amplified polymorphic DNA (RAPD), amplified fragment length polymorphism (AFLP), and inter simple sequence repeat (ISSR); however, these markers have various deficiencies, such as poor stability, poor repeatability, heavy workload, and high sample quality requirements (Clarkson et al., 2013; Tok et al., 2016; Song et al., 2021). Microsatellite loci or simple sequence repeats (SSRs) are PCR-based molecular markers that may be more desirable for population genetic analysis as they enable simpler retrieval of accurate polymorphic data due to codominance, high polymorphism and high information content (Li et al., 2008). SSRs have been employed to characterize plant pathogen population from a spectrum of hosts in different geographical areas. For example, Luo et al. (2015) developed SSR markers to assess the genetic variation among the wheat stripe rust fungus population. Similarly, the genetic variation of *S. sclerotiorum* isolates from sunflower in Northeast China was analyzed using SSRs (Liu et al., 2018). According to the different original sequences of SSR, it can be divided into genomic-SSRs (gSSRs) and expressed sequence tag-SSRs (EST-SSRs). Compared with gSSR, EST-SSR has the advantages of simple steps, low cost, and more evolved. Therefore, EST-SSR is an economical and effective DNA molecular marker. However, EST-SSRs have not been used to examine the populations *S. rolfsii* adapted to different hosts.

In this study, we comprehensively analyzed the genetic diversity of 120 strains from various hosts and diverse geographical locations in China based on genotyping with EST-SSR markers. Using the transcriptome data of *S. rolfsii*, the EST-SSRs markers were developed, and the polymorphisms among different populations were analyzed. The findings of this study improve current knowledge on the population structure and genetic diversity of *S. rolfsii*, specifically in Chinese herbal medicine from China, and may provide valuable information for the prevention and control of the disease and the breeding of resistant varieties.

## 2. Materials and methods

### 2.1. Fungal isolation

Plants with southern blight disease were collected from 13 hosts in 7 provinces from 2017–2021 (Supplementary Table S1; Supplementary Figure S1). The fungus from the infected sample was obtained and purified via hyphal tip isolation on PDA (Potato Dextrose Agar) plates supplemented with 50 μg/mL teracycline antibiotics to prevent bacterial contamination. The isolates were stored at 4°C for a future study. Morphological identification of the isolates was based on the cultural characteristics of *S. rolfsii* and morphology of the mycelial mat and sclerotium formation traits.

### 2.2. DNA extraction

*S. rolfsii* isolates on PDA medium were stored in a BOD incubator at 28°C for 7 days. The mycelial mat was harvested via scraping with a sterile spatula and immediately frozen in liquid nitrogen. After freeze-drying, fungal mycelia were ground under liquid nitrogen using a mortar and pestle. Genomic DNA was extracted using a Fungal DNA isolation kit (Tsingke Biotechnology Co. Ltd) according to the producer’s protocol. The DNA was quantitatively and qualitatively determined using NanoDrop (Themo Scientific), diluted to a working concentration of 20 ng/μl, and stored in aliquots at −20°C until further use.

### 2.3. PCR amplification

To verify the species identity of some of the newly collected isolates, universal ITS primers with ITS1 as forward and ITS4 as reverse primer and *LSU* primers with NL1 as forward and NL4 as reverse primer were used for PCR amplification of the 120 isolates (White et al., 1990; Table 1). The reaction volume of 25 μL comprised 12.5 μL of 10× PCR buffer, 10 μM forward and reverse primers, 1 μL of genomic DNA, and 9.5 μL ddH_2_O and PCR was performed in a thermal cycler (Biometra, ILS, United States). The cycling program was as follows: initial denaturation at 95°C for 4 min followed by 35 cycles of denaturation at 94°C for 45 s, optimized annealing temperature for 45 s and extension at 72°C for 1 min, and a final extension at 72°C for 10 min. The PCR amplicons were electrophoresed on 1% agarose gel in 1× TBE buffer (89 mM Tris–HCl, 89 mM boric acid, 2 mM EDTA and pH 8.35) and visualized in a UV transilluminator.

**Table 1:** Information of the primers used for *ITS* and *LSU* amplification.

Target	Primer	Sequence(5′ to 3′)	T(°C)	Reference
ITS	ITS4	TCCTCCGCTTATTGATATGC	56	White et al., 1990
	ITS5	GGAAGTAAAAGTCGTAACAAGG		
*LSU*	NL1	GCATATCAATAAGCGGAGGAAAAG	57	
	NL4	GGTCCGTGTTTCAAGACGG		

### 2.4. RNA extraction, library construction, and sequencing

Samples from three periods, namely the mycelium stage (S1), sclerotia of early development stage (S2) and mature sclerotia (S3) stage, were collected from strain CB1 of *Macleaya cordata* in Hunan Province. The collected samples were immediately frozen in liquid nitrogen and stored at −80°C for further use. Total RNA was extracted according to the manufacturer’s instructions for the Trizol reagent kit (Life Technologies inc, United States). The mRNA of each sample that met the quality requirements was enriched by Oligo (dT) beads. Then the enriched mRNA was fragmented into short fragments using fragmentation buffer and reverse transcript into cDNA with random primers. Second-strand cDNA were synthesized by DNA polymerase I, RNase H, dNTP and buffer. Then the cDNA fragments were purified with 1.8X Agencourt AMPure XP Beads, end repaired, poly(A) added, and ligated to Illumina sequencing adapters. The ligation products were size selected by agarose gel electrophoresis, PCR amplified, and sequenced using Illumina HiSeq™ 4,000 by Gene Denovo Biotechnology Co (Guangzhou, China). Transcriptome *de novo* assembly was carried out with short reads assembling program – Trinity (Grabherr et al., 2011).

All raw sequencing data were submitted to the Genome Sequence Archive (GSA) database under the BioProject accession number CRA009668 at the BIG Sub website.^1^

### 2.5. Detection of The EST-SSRs loci and primer design

The detection and localization of potential SSRs were searched and implemented from 25,185 unigenes using the MISA tool.^2^ The number, frequency, maximum repeat motif, and percentage of different repeat types of SSR were identified in the *S. rolfsii* transcriptome. Primer 6.0 software was used to design the primers for the SSR locus. The main selection parameters for screening the primers were as follows: I. sites with repetition units of 2, 3, 4, and 5 bases with only one repetition type; II. fragment length greater than 150 bp and less than 300 bp; III. location of genes not markedly concentrated, preferential selection of the loci with polymorphism, and even selection of the duplicate units of different combinations; IV. primers with length between 20 and 23 bp and TM value of approximately 60°C; V. primers with base repeats less than or equal to 4; VI. 5′ and 3′ ends do not contain two consecutive A/T bases; and VII. no repeat sequence in the primer.

### 2.6. Primer validation and polymorphism detection

To validate the quality of the primer, 200 primer pair sequences were randomly selected and synthesized by Tsingke Biotechnology Co., Ltd. (Beijing, China) and used for PCR amplification. The reaction volume of 20 μL comprised 10 μL 2 × TSINGKE Master mix (Tsingke Biotechnology Co., Ltd. Beijing, China), 1 μL forward primer (connector “CAG,” 10 mM), 1 μL reverse primer (10 mM), 1 μL genomic DAN (20 ng/μL), and 7 μL ddH_2_O. The PCR amplification conditions were as follows: 94°C for 5 min, followed by 35 cycles at 94°C for 30 s, 60°C for 30 s, 72°C for 30 s, and then a final extension at 72°C for 5 min, using a Thermal Cycler (Hangzhou Bioer Technology Co., Ltd., Hangzhou, China). All primer pairs were used for amplification of 8 samples with obvious morphological differences to select the primers that resulted in clear amplification bands. Then 16 samples from different hosts and different geographical sources were used as templates, and primers with good polymorphism were screened by capillary electrophoresis.

The obtained PCR products were detected by capillary electrophoresis with 3,730 XL sequencer (ABI 3730xl Genetic analysis instrument). Primers with high specificity, good polymorphism, and good repeatability were screened by analyzing the locus peak and data information. To clarify the polymorphism of EST-SSR primers and populations, the number of alleles (Na), effective number of alleles (Ne), Shannon index (I), polymorphism information content (PIC), and observed (Ho) and expected (He) heterozygosity were calculated using Popgene 1.32 and GenAlEx 6.501 (Peakall and Smouse, 2012).

### 2.7. Population genetic diversity

The obtained EST-SSR primers were used to analyze the population genetic diversity of 120 strains from 22 populations. To determine the genetic variation between geographic populations and host populations, including within populations, among populations, within groups, and among groups, the analysis of molecular variance (AMOVA) function in Gene AlEx 6.501 was applied. The genetic structure of 7 geographic populations was analyzed using Structure 2.3.4 based on Bayesian analysis. Twenty independent runs were performed for each K value (Evanno et al., 2005). The burn-in period iterations and Markov chain Monte Carlo repetitions for each run (K ranged from 2 to 10) were 100,000 each. The results obtained from Structure were further analyzed using the software of Structure Harvest^3^ to obtain the best K value. Unweighted pair group (UPGMA) in Populations-1_2_30 was used to cluster the populations of *S. rolfsii*, and a dendrogram was generated from this clustering.

## 3. Results

### 3.1. Illumina sequencing and *de novo* assembly

The whole RNA was, respectively, extracted from the samples as following: *S. rolfsii* mycelium stage (S1), sclerotia of early development stage (S2) and mature sclerotia (S3) stage, and then sequenced by Illumina technology. After filtering, 27,173,090, 26,896,318 and 22,913,500 clean reads were obtained from S1 stage, 24,936,756, 23,481,220 and 27,038,696 clean reads were obtained from S2 stage, 27,164,438, 28,988,848 and 25,025,724 clean reads were obtained from S3 stage, respectively. All of these clean reads with total 36,165,475 assembled bases were clustered into 28,158 unigenes, ranged from 201 bp to 16,402 bp on the length, of which the average length was 1,284 bp. The GC percentage was 48.29% and the N_50_ was 2,379. Of these, 3,299 SSR sequences were detected, including 579 unigenes containing multiple SSRs (Table 2). In general, the SSR sequence appeared at an average interval of 15.43 kB, and the frequency of SSR was 0.0648 SSR/kB.

**Table 2:** SSR type statistics of *Sclerotium rolfsii.*

Stat Item	Number
Total number of sequences examined	28,158
Total size of examined sequences (kb)	36,165
Total number of identified SSRs	3,299
Number of SSR containing sequences	2,344
Number of sequences containing more than 1 SSR	579
Number of SSRs present in compound formation	198

### 3.2. Frequency and distribution of SSRs in the unigenes

The proportions of five different SSR unit types were not evenly distributed across all SSRs. The frequency of the occurrence of different repeat types was as follows: 52.53% for tri-repeats and 31.56% for di-repeats, ultimately accounting for the highest proportions. In addition, the frequencies of tetra-repeats, penta-repeats, and hexa-repeats were 10.28, 2.67, and 2.97%, respectively. Of the 1,041 di-repeats motifs, the (AG/CT)_n_ di-repeats motifs were the most abundant. The other four major unit types included (ATC/ATG)_n_ in tri-repeats, (AAAG/CTTT)_n_ in tetra-repeats, (AAAAG/CTTTT)_n_ in penta-repeats, and (AAGGAG/CCTTCT)_n_ and (ACCAGC/CTGGTG)_n_ in hexa-repeats, with frequencies of 11.91, 3.27, 0.61, and 0.15%, respectively (Table 3).

**Table 3:** Frequencies of the different repeat types of SSR in the *Sclerotium rolfsii* transcriptom.

Repeat type	Number	Frequency (%)	Maximum repeat motif, numbei, and percentage
Di-repeats	1,041	31.56	AG/CT; 579; 17.55%
Tri-repeats	1733	52.53	ATC/ATG; 393; 11.91%
Tetra-repeats	339	10.28	AAAG/CTTT; 108; 3.27%
Penta-repeats	88	2.67	AAAAG/CTTTT; 20; 0.61%
Hexa-repeats	98	2.97	AAGGAG/CCTTCT, ACCAGC/CTGGTG; 5; 0.15%

**Figure 1:**
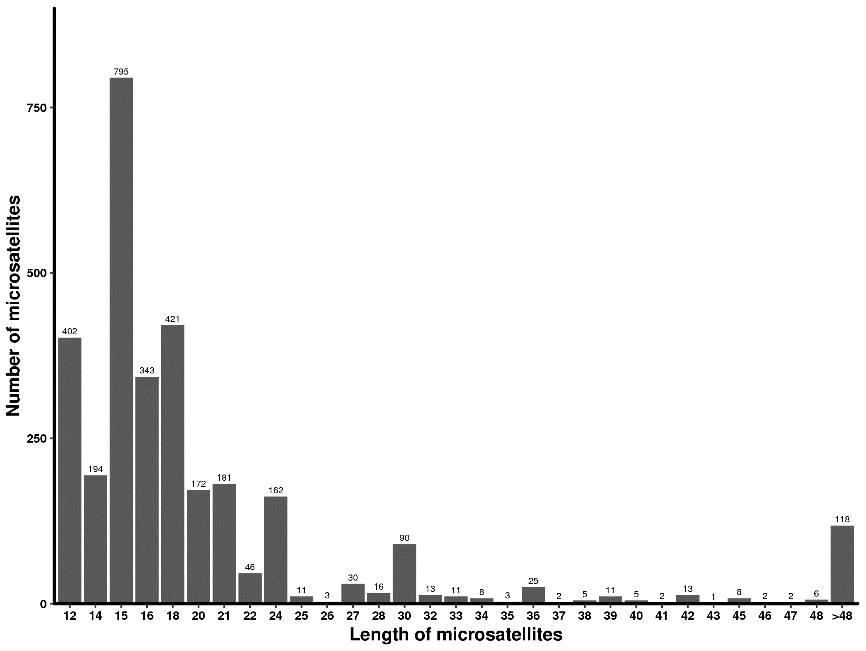
Length distribution and number of SSR in the *Sclerotium rolfsii* transcriptome.

The SSR length of strain CB1 ranged from 12 to 212 bp, with an average length of 72.78 bp. Most microsatellite sequences were 12–36 bp in length, accounting for 39.46% of the total microsatellite length distribution. There were 175 microsatellite sequences longer than 36 bp. The number of microsatellite sequences of 15 bp was the largest, accounting for 15.96% of the total number, followed by 14 bp and 20 bp microsatellite sequences, accounting for 12.96 and 13.58%, respectively (Figure 1).

### 3.3. Development and validation of novel EST-SSRs

Primer 6.0 software was used to design primer pairs that meet the required criteria. Briefly, we randomly selected and synthesized 200 primer pairs, and initially tested the polymorphism of SSR primers (Supplementary Table S2). Thirty-six primer pairs with polymorphism were separated via agarose gel electrophoresis. The results of polymorphism analysis of loci detected by capillary electrophoresis showed that 9 primer pairs had good polymorphism for genetic diversity, including BJ015, BJ071, BJ082, BJ112, BJ130, BJ152, BJ156, BJ157, and BJ184 (Table 4; Supplementary Figures S2, S3).

**Table 4:** Information for the nine pairs of SSR primers.

Primer	Forward Primer sequence	Repeated bases	Times of repetition
BJ015	F: 5’-AGAAGACGGGAATAAGCCGT-3′ R: 5’-AGTTGATTGGGCAAACTTGG-3’	TGG	6
BJ071	F: 5’-GTCACGCAATTGGAACACAC-3′ R: 5’-GAGAGCGGACACTCTCCAAG-3’	GAT	5
BJ082	F: 5’-ATCCAAATCAAGCATCTGGC-3′ R: 5’-GATGTACGGGTCGTATTCGG-3’	CTG	6
BJ112	F: 5’-GGGAGGATGCAAGAACTGAA-3′ R: 5’-ATTTATACCCGCTCACACCG-3’	GT	6
BJ130	F: 5’-TGGCTCTCTACGGACGAACT-3′ R: 5’-TATCATCGCCTTCCTTGGAC-3’	CT	17
BJ152	F: 5’-CCGAGAGAGCAAGAGAATGG-3′ R: 5’-AAAGGCCTGCTCGACTTGTA-3’	GGAG	8
BJ156	F: 5’-TGATCGGGTAGGTGAGGTTC-3′ R: 5’-CGTTACATACGGCCAGTCCT-3’	GAT	5
BJ157	F: 5’-TGCTCTTGTCCAGTGGAGTG-3′ R: 5’-GGTTGCCTTTCCCTTCTCTC-3’	GT	6
BJ184	F: 5’-CACACAAGGGAGCACAGAGA-3′ R: 5’-GGCAACAACGCTCATCAGTA-3’	AAT	5

### 3.4. Polymorphism of the SSR loci

Sixty-six alleles (*Na*) were detected in the 22 populations at an average of 7.333 alleles/locus. BJ184 was the locus with the largest number of alleles (11). The values for *Ne*, He, and *PIC* were 1.963, 0.493, and 0.477, respectively. The value of *Ne* was in the range of 1.963 and 5.993, with an average of 3.370. BJ112 had the highest value of *I* (1.981) and BJ071 had the lowest value of *I* (1.044), with an average value of 1.414. The value of *He* ranged from 0.493 to 0.837, with a mean of 0.671. The value of *PIC* varied from 0.477 to 0.814, with an average of 0.630. For the overall *PIC*, one locus had values between 0.25 and 0.50 (moderately informative), while BJ015, BJ071, BJ082, BJ112, BJ130, BJ152, BJ156, and BJ157 had values >0.50 (highly informative, Table 5). Overall, the 9 pairs of SSR primers developed in this study had high polymorphisms, and can be used to analyze the genetic diversity of *S. rolfsii*.

**Table 5:** Polymorphism of the 9 pairs of SSR primers.

Locus	*Na*	*Ne*	*I*	*PIC*	*Ho*	*He*
BJ015	4	2.8338	1.0969	0.5734	0.0917	0.6498
BJ071	4	2.4492	1.0435	0.5178	0.6167	0.5942
BJ082	6	3.6966	1.4943	0.6908	0.2583	0.7325
BJ112	10	5.9925	1.9814	0.8140	0.3917	0.8366
BJ130	9	3.8191	1.6026	0.7046	0.2000	0.7412
BJ152	8	4.2013	1.7293	0.7387	0.3667	0.7652
BJ156	6	2.1467	1.1129	0.5054	0.1833	0.5364
BJ157	8	3.2301	1.4558	0.6518	0.1750	0.6933
BJ184	11	1.9625	1.2087	0.4767	0.0417	0.4925
Mean	7.3333	3.3702	1.4139	0.6303	0.2583	0.6713
St. Dev	2.5000	1.2505	0.3232		0.1763	0.1128

Na, No. of different Alleles; Ne, No. of effective alleles; I, Shannon’s Information Index; PIC, Polymorphic information index; Ho, Observed Heterozygosity; He, Expected Heterozygosity.

### 3.5. Genetic diversity analysis

The genetic diversity of the *S. rolfsii* population was assessed based on the geographical origin, i.e., Anhui group, Guangxi group, Henan group, Hubei group, Hunan group, Sichuan group, and Shandong group (Table 6). The diversity indices, including Na (average number of alleles), Ne (number of effective alleles), I (Shannon’s Information Index), Ho (Observed Heterozygosity), He (Expected Heterozygosity), and F (Fixation Index), were calculated. The Ne value among 7 populations ranged from 1.231 to 3.281, and the highest and lowest levels of the Ne value were found in Henan and Hunan, respectively. The ranking was the same with the Shannon diversity index (I). Comprehensive analysis of the genetic diversity index revealed that the genetic diversity index of the Henan and Shandong populations was low. The low diversity within the Shandong group is most likely attributable to the smaller population size (four isolates). More significant diversity was observed in larger populations from Hunan (49 isolates) province.

**Table 6:** The genetic diversity of *Sclerotium rolfsii* in different regions.

Populations	Number	Na	Ne	I	Ho	He	F
Anhui	10	3.778	2.989	1.115	0.344	0.617	0.502
Guangxi	13	4.000	2.761	1.104	0.427	0.589	0.317
Henan	11	1.333	1.231	0.174	0.222	0.120	−0.627
Hubei	28	3.778	2.199	0.935	0.202	0.503	0.627
Hunan	49	5.667	3.281	1.290	0.227	0.629	0.650
Sichuan	5	2.889	2.205	0.857	0.400	0.509	0.324
Shandong	4	1.333	1.276	0.213	0.194	0.149	−0.200
Mean	17.143	3.254	2.277	0.813	0.288	0.445	0.378

Na, No. of different Alleles; Ne, No. of effective Alleles; I, Shannon’s Information Index; Ho, Observed Heterozygosity; He, Expected Heterozygosity; F, Fixation Index.

The genetic diversity of the *S. rolfsii* population was also assessed based on the host (Table 7). The estimate of the Ne value was lowest for the *Amorphophallus rivieri* group and highest for the *Dendrobium nobile* group. The ranking was the same with the Shannon diversity index (I). The highest and lowest levels of diversity were found in *D. nobile* and *A. rivieri*, respectively. The fixation index (F) of the geographical population and host population were significantly greater than or less than 0, indicating the absence of heterozygotes or homozygotes.

**Table 7:** The genetic diversity of different host populations.

Populations	Number	Na	Ne	I	Ho	He	F
*M. cordata*	20	3.667	2.732	1.054	0.161	0.585	0.738
*C. glaucescens*	6	2.556	2.037	0.704	0.148	0.414	0.602
*P. ternata*	17	4.000	2.359	1.015	0.209	0.552	0.636
*A. macrocephala*	3	1.333	1.286	0.189	0.222	0.123	−0.818
*P. depressa*	2	1.889	1.726	0.510	0.222	0.333	0.289
*P. sibiricum*	21	3.778	2.131	0.917	0.317	0.501	0.434
*C. chinensis*	5	2.778	2.145	0.809	0.422	0.484	0.182
*A. hypogaea*	15	1.444	1.247	0.197	0.215	0.132	−0.365
*A. konjac*	5	1.222	1.222	0.154	0.222	0.111	−1.000
*O. sativa*	6	2.778	2.085	0.803	0.463	0.477	0.107
*D. nobile*	10	3.778	2.989	1.115	0.344	0.617	0.502
*S. ningpoensis*	5	1.556	1.471	0.357	0.111	0.251	0.600
*H. tuberosus*	5	2.333	1.902	0.636	0.467	0.393	−0.195
Mean	9.231	2.547	1.949	0.651	0.271	0.383	0.305

Na, No. of different Alleles; Ne, No. of effective Alleles; I, Shannon’s Information Index; Ho, Observed Heterozygosity;He, Expected Heterozygosity; F, Fixation Index.

### 3.6. Analysis of molecular variance (AMOVA)

The seven populations were analyzed to determine the genetic variation among and within populations using AMOVA (Table 8). Based on the analysis, 28% variation was observed among the population and 72% was observed within the population. A variation was observed among geographical populations and within populations, and the variation within populations was 2.5-fold that between populations. The genetic variation was mainly derived from within populations. The analysis of gene flow and genetic differentiation between the two populations showed that the gene flow value ranged from 0.055 (Sichuan/Henan) to 10.254 (Hunan/Anhui), and the genetic differentiation value ranged from 0.024 (Anhui/Hunan) to 0.819 (Henan/Sichuan; Table 9). Overall, gene flow was frequent and the variation was not obvious in Anhui Province and Hunan Province, while the gene flow value was small and genetic variation was large in Sichuan Province and Henan Province, which might be related to the geographical difference.

**Table 8:** AMOVA of the populations in different regions.

Source	df	SS	Est. Var.	Percentage %
Among Pops	6	306.408	2.902	28%
Within Pops	113	858.575	7.598	72%
Total	119	1164.983	10.500	100%

**Table 9:** Gene flow (above the diagonal) and genetic differentiation (below the diagonal) results of populations in different regions.

Populations	Anhui	Guangxi	Hubei	Henan	Hunan	Sichuan	Shandong
Anhui		0.534	1.192	0.185	10.254	0.659	0.358
Guangxi	0.319		0.388	0.117	0.612	0.680	0.197
Hubei	0.173	0.392		0.580	2.313	0.352	0.938
Henan	0.574	0.681	0.301		0.418	0.055	1.170
Hunan	0.024	0.290	0.098	0.374		0.821	0.562
Sichuan	0.275	0.269	0.415	0.819	0.233		0.130
Shandong	0.411	0.559	0.210	0.176	0.308	0.657	

The 13 *S. rolfsii* clusters were further analyzed using hierarchical AMOVA and pairwise genetic differentiation. In the AMOVA test, total genetic variation was partitioned at two levels: among clusters and among isolates within clusters. This partition revealed that 39% of the total genetic variation was due to genetic differences among clusters, while the remnant and larger source of diversity (61%) was located among isolates within clusters (Table 10). Further analysis of gene flow and genetic differentiation between the two populations revealed that the gene flow values ranged from 0.003 (*A. rivieri*/*A. macrocephala*) to 17.531 (*S. ningpoensis*/*P. ternata*), and the genetic differentiation values ranged from 0.014 (*S. ningpoensis*/*P. ternata*) to 0.990 (*A. rivieri*/*A. macrocephala*), except for the *A. rivieri*/*A. hypogaea* and *P. ternata*/*C. glaucescens* groups (Table 11). No genetic differentiation existed between *A. rivieri* and *Arachis hypogaea*, and *Pinellia ternata* and *Cynanchum stauntonii*. In contrast, the genetic differentiation between *Scrophularia ningpoensis* and *P. ternata* was relatively low, and that between *A. rivieri* and *Atractylodes macrocephala* was relatively obvious. The results were not obviously related to the host, but were related to the geographical origin of the host population.

**Table 10:** AMOVA of the populations of different host populations.

Source	df	*SS*	Est. Var.	Percentage variation
Among Pops	12	501.643	4.018	39%
Within Pops	107	663.340	6.199	61%
Total	119	1164.983	10.217	100%

**Table 11:** Gene flow (above the diagonal) and genetic differentiation (below the diagonal) results of different host populations.

Populations	*M.cordata*	*C.glaucescens*	*P.ternata*	*A.macrocephala*	*P.depressa*	*P.sibiricum*	*C.chinensis*	*A.hypogaea*	*A.rivieri*	*O.sativa*	*D.nobile*;	*S.ningpoensis*	*H.tuberosus*
*M.cordata*		2.400	3.101	0.489	0.593	0.633	0.668	0.288	0.455	0.701	1.871	1.096	0.574
*C.glaucescens*	0.094		916.241	0.364	0.375	0.628	0.391	0.126	0.253	0.267	2.046	8.421	0.205
*P.ternata*	0.075	0.000		0.525	0.507	0.638	0.519	0.293	0.486	0.466	1.462	17.531	0.371
*A.macrocephala*	0.338	0.407	0.323		0.097	0.644	0.139	0.011	0.003	0.089	0.593	0.122	0.055
*P.depressa*	0.296	0.400	0.330	0.720		0.320	0.260	0.023	0.038	0.192	0.568	0.167	0.132
*P.sibiricum*	0.283	0.285	0.281	0.280	0.439		0.613	0.147	0.212	0.226	2.093	0.321	0.189
*C.chinensis*	0.272	0.390	0.325	0.643	0.490	0.290		0.059	0.108	0.267	0.830	0.210	0.182
*A.hypogaea*	0.464	0.666	0.460	0.959	0.916	0.629	0.810		9999.000	0.048	0.161	0.060	0.025
*A.rivieri*	0.355	0.497	0.340	0.990	0.867	0.541	0.699	0.000		0.083	0.290	0.104	0.039
*O.sativa*	0.263	0.484	0.349	0.739	0.566	0.526	0.483	0.839	0.752		0.436	0.170	2.264
*D.nobile*	0.118	0.109	0.146	0.296	0.306	0.107	0.232	0.608	0.463	0.364		0.707	0.341
*S.ningpoensis*	0.186	0.029	0.014	0.671	0.599	0.438	0.544	0.806	0.707	0.595	0.261		0.118
*H.tuberosus*	0.303	0.550	0.403	0.821	0.655	0.569	0.579	0.908	0.865	0.099	0.423	0.679	

### 3.7. Population structure

**Figure 2:**
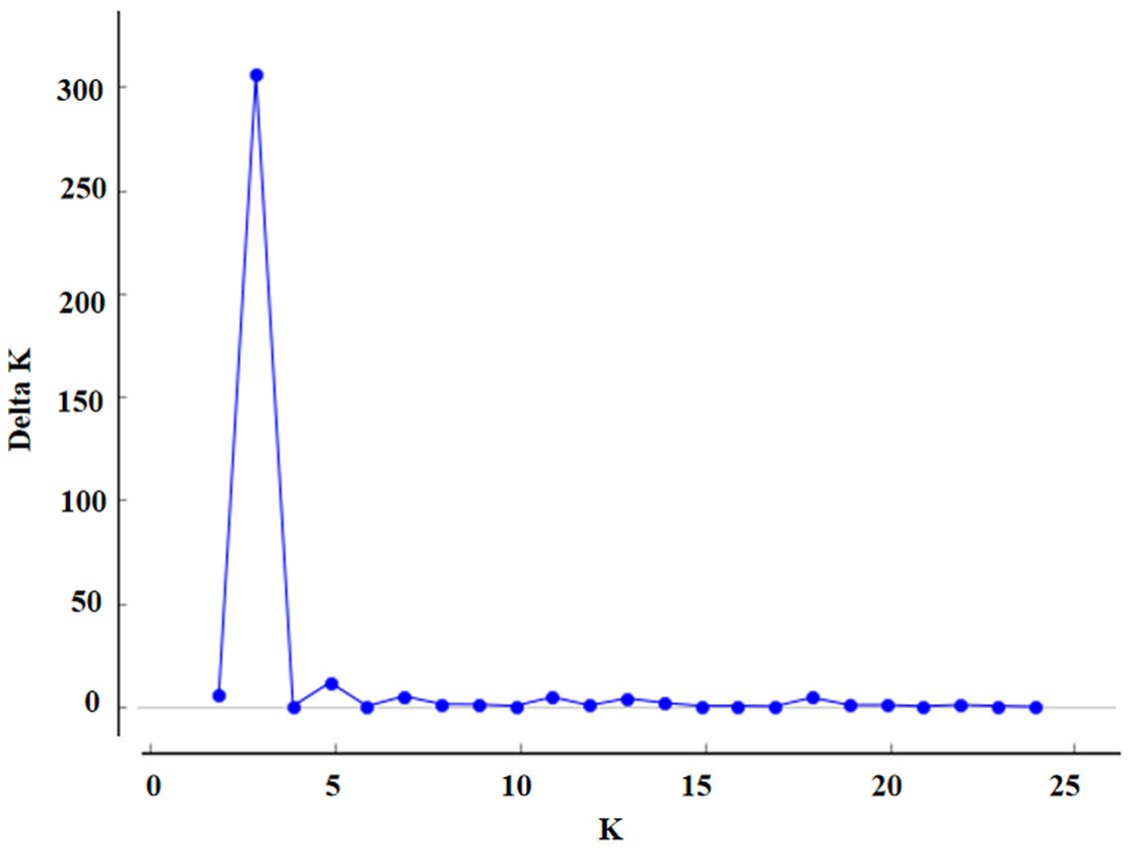
The magnitude of Delta K at each level of K.

**Figure 3:**
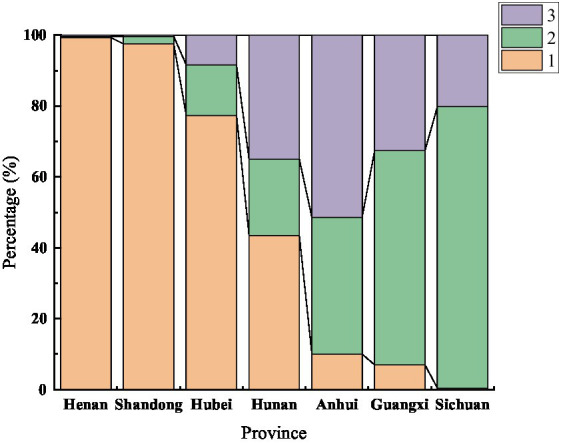
Structure results of the 7 populations (K = 3).

Based on AMOVA, the differences between groups were mainly related to geographical location. The population structure of 7 geographical populations was analyzed. A clustering pattern was identified via STRUCTURE analysis. Using △K, which is an *ad hoc* statistic based on the rate of change in the log probability of data between successive K values (Evanno et al., 2005), a K value of 3 was identified, which suggested that the isolates were broadly divided into three populations (Figures 2, 3). Population 1 mainly comprised isolates from Henan, Shandong, and Hubei; population 2 mainly comprised isolates from Sichuan and Guangxi; and population 3 mainly comprised isolates from Anhui. The distribution of gene in each region is consistent with the geographical location.

**Figure 4:**
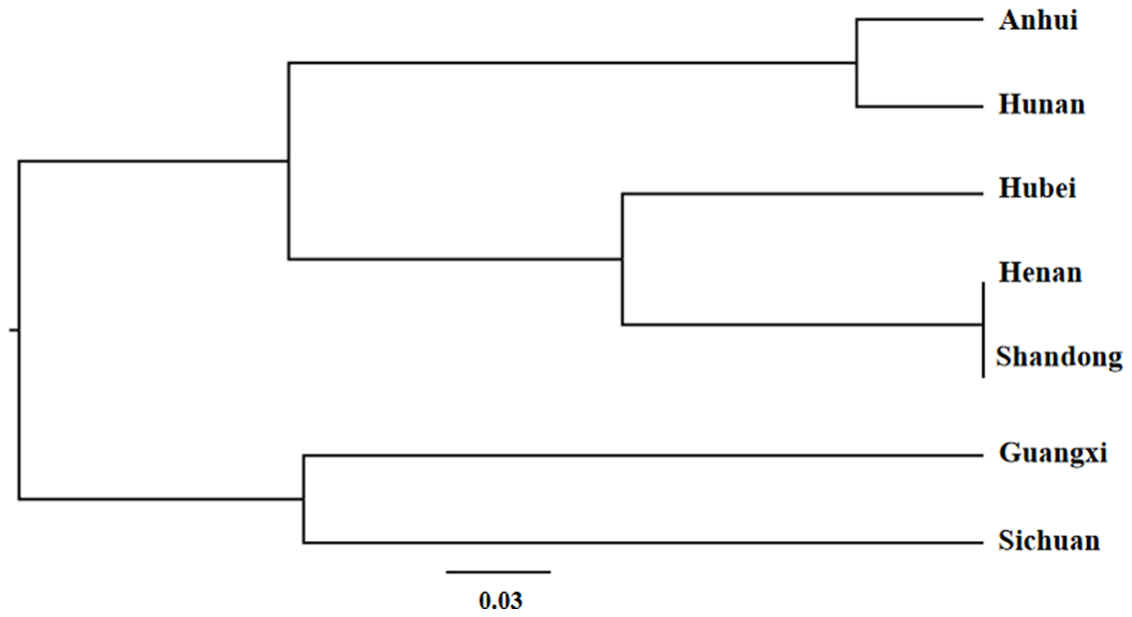
The UPGMA tree of the 7 populations.

Based on the genetic distance of Nei, cluster analysis of the 7 populations were performed. Using UPGMA cluster analysis, the populations were grouped into two main clusters (Figure 4). Cluster A contained 5 populations, *viz.* Anhui, Hunan, Hubei, Henan, and Shandong, of which the first two and last three formed two separate sub-clusters. Cluster B contained two populations, *viz.* Guangxi and Sichuan.

## 4. Discussion

*S. rolfsii*, which causes root rot disease, is considered a serious problem affecting crop cultivation in contaminated areas due to its wide host range, genetic population diversity, and its ability to produce abundant resistant sclerotia. This disease causes severe damage during all stages of crop growth. Further, significant yield reduction caused by *S. rolfsii* infection has been reported in areas that grow various Chinese medicinal materials, such as in Hubei, Hunan, and Anhui provinces in China, with infection rates ranging from 5–42% (You et al., 2016; Nie et al., 2020; Zhang et al., 2020; Cheng et al., 2022). To our knowledge, the presence of *S. rolfsii* as a causal agent of southern blight disease on some Chinese medicinal materials was first revealed in this study. Owing to the high occurrence in these areas and lack of information on *S. rolfsi*i population in this circumscribed area, we aimed to examine the *S. rolfsii* population from different hosts in China and locations, such as Hubei, Huan, Henan, Shandong, Anhiu, Guangxi, and Sichuan provinces. The present investigation revealed the molecular variability among the 120 isolates of *S. rolfsii* collected from 13 host crops belonging to various locations in 2017–2021.

The genetic diversity of fungal pathogens is essential for assessing the characteristics and diversity of fungal pathogens and would be helpful for the development of an effective integrated disease management strategy. Molecular markers play a major role in analyzing the genetic variation of fungal pathogens (Zhang et al., 2014). In some studies, the molecular genetic diversity of the *S. rolfsi*i isolates obtained from different hosts and/or geographical regions was tested using RAPD, ISSR, and other molecular means. Almeida et al. (2010) collected 30 *S. rolfsii* isolates from different hosts in Brazil and found significant genetic variability among isolates using RAPD analysis. During same period, Punja and Sun (2001) collected 132 *S. rolfsii* isolates from 13 countries and found high level of variations among samples based on RAPD analysis. Jebaraj et al. (2017) used RAPD and ISSR to assess the genetic diversity and population structure of 22 *S. rolfsii* isolates. Recent advances in large-scale RNA-seq have provided a fast, cost-effective, and reliable approach to generate large expression datasets in non-model species, thereby markedly accelerating the development of microsatellites (Bhandawat et al., 2016; Lei et al., 2017). Currently, RNA-seq has been used in the SSR development of *G. yamadae* and *G. asiaticum* (Tao et al., 2018). EST-SSR can detect numerous alleles, with high repeatability and inter-species transferability due to the presence of microsatellites regions throughout the transcriptome (Weng et al., 2020). In addition, EST-SSR can display the functional information of the sample genome, and the sequences identified in the homologous genes are more conserved, so it has the characteristics of large amount of information, good universality and simple development (Wu et al., 2014). EST-SSR is also an effective alternative for studying genetic variability, bio-effective populations, genome identification, kinship, etc. (Clauss et al., 2002; Hisano et al., 2007). To our knowledge, a study of *S. rolfsii* on different hosts and geographical regions using EST-SSR has not been previously performed. In the present study, 9 pairs of polymorphic primers were screened from 200 pairs of primers for *S. rolfsii* based on RNA-seq data and capillary electrophoresis detection technique. These methods were effective and cost saving, indicating the effective and feasible screening of EST-SSR primers using the sequencing method.

To obtain suitable EST-SSR primers for *S. rolfsii* with different morphological characteristics, such as mycelia growth rate and sclerotia, we collected and combined mycelia from three growth stages of the strain CB1 for RNA extraction. Transcriptomic SSR locus analysis revealed that tri-nucleotide repeats had the highest occurrence frequency, accounting for 52.53% of the repeats, which aligns with the results of previous reports on other fungi (Qian et al., 2013). However, tetra-nucleotide repeats tend to stutter less than tri-nucleotide and di-nucleotide repeats and are markedly more accurate and reliable (Weng et al., 2020). Therefore, one of the principles in selecting EST-SSR primers of *S. rolfsii* involved the selection of a sequence with a repeat unit of di- to penta-nucleotides to increase diversification. Among the nine selected primers, the tri-nucleotide repeat type accounted for the highest proportion of 55.56%, followed by di-nucleotides and tetra-nucleotides, which accounted for 33.33 and 11.11%, respectively. Therefore, EST-SSRs primers containing tri-nucleotide and di-nucleotide repeats are more suitable for this study, possibly due to species. Broquet et al. (2007) proposed that the number of microsatellite markers has an important implication for all subsequent analyses. Too many markers may increase genotyping errors and false genotypes, and overestimate the population size; however, too few or insufficient markers may lead to underestimation. In the nine EST-SSRs primers, 5–6 repetitions accounted for the majority (77.78%).

A total of 66 allelic loci were detected by the 9 primers in the 120 strains. The mean value of the Shannon index (I) was 1.4139, while that of the polymorphic information index (PIC) was 0.6303, indicating good polymorphism with *S. rolfsii*. Due to the large number of *S. rolfsii* isolates and the confusion of host and geographical origin in this study, we divided the isolates into 7 geographic populations and 13 host populations for genetic diversity analysis. Comprehensive analysis of the genetic diversity index revealed that the genetic diversity of the Henan populations was low, and the Hunan population had the highest diversity. This result might be due to the incomparability of host species and population size among these groups (*n* = 49 for Hunan, *n* = 11 for Henan, and 4 hosts for Hunan, 1 host for Henan). Comprehensive analysis of the genetic diversity index of the host populations revealed that *A. rivieri* had the lowest genetic diversity index and *D. nobile* had the highest genetic diversity index. The results of the present study align with those of Gupta et al. (1994), Rathour et al. (2004), and Mishra et al. (2006) where a significant level of genetic variations among fungal pathogens was found. Genetic variation and the stability of genetic diversity are crucial to the evolutionary potential and long-term interest of any species, whose estimates are a major component of conservation studies (Lozier, 2014).

AMOVA analysis showed that genetic differentiation was more related to geographical location than to the host. A variability study of *S. rolfsii* based on mycelial compatibility, rDNA, RAPD, and AFLP is commonly used to understand the distribution pattern of populations within and between different geographical regions (Punja and Sun, 2001; Adandonon et al., 2005). Early studies involving MCG analysis of *S. rolfsii* suggested a correlation between MCG and the geographical source of isolates, which is relatively similar to the results of this study (Remesal et al., 2012). Akram et al. (2015) collected 20 isolates of *S. rolfsii* from different host plants and various geographical locations to carry out genetic diversity analysis based on RAPD. Their results revealed no correlation between cultural, morphological characters, origin of hosts, and geographical location with molecular diversity. In a similar study, Song et al. (2021) found no significant correlation between genetic diversity and geographical origin using 39 *S. rolfsii* strains from 12 provinces in China based on ISSR and ITS-RFLP genetic analysis. Jebaraj et al. (2017) also showed that the nature and type of host may influence genetic variation between the isolates using nuclear (RAPD & ISSR) markers. Of note, all diversity indices are affected by sample size (Barrantes and Sandoval, 2009; Weng et al., 2020). Thus, the above results will be affected by the low sample size of each group. More detailed studies must be performed to confirm this relationship and its overall significance.

Based on AMOVA, STRUCTURE analysis of the seven geographic populations revealed that they can be divided into three groups. STRUCTURE analysis revealed three gene banks in the seven populations. The distribution of gene banks in each population was related to the geographical location. Cluster analysis showed that the closely spaced populations grouped in one cluster (sub-cluster A2 and cluster B), suggesting a correlation between the genetic and geographical location of the population. The sampling point of Anhui is located at the junction of Anhui and Hubei, and Hubei and Hunan are adjacent to each other with frequent exchanges. As a result, Anhui and Hunan belonged to one branch in the clustering results. The limited number of specimens may also be the reason for the results. The sample size should be sufficiently large to ensure the results reflect the true ecological patterns that could otherwise lead to contingencies.

## 5. Conclusion

In this study, 120 *S. rolfsi*i isolates from 13 hosts in 7 provinces of China were isolated, purified, and identified. Based on the transcriptional sequencing of strain CB1, the SSR loci were analyzed and nine EST-SSR primers with good polymorphism were developed. To our knowledge, this is the first use of the EST-SSR method to analyze the genetic diversity of *S. rolfsii*, especially from areas that grow Chinese medicinal materials in China, which revealed the existence of broader genetic variations among the pathogen. Such finding will facilitate the development of effective disease management strategies based on molecular breeding and other advanced approaches. The results of population STRUCTURE analysis were consistent with those of geographical location, which preliminarily proved that geographical location had a certain correlation with the genetic diversity of pathogen. In future studies, we will collect more *S. rolfsi*i specimens from more regions and hosts. Using larger datasets should better reflect the distribution and genetic status of *S. rolfsi*i.

## Data availability statement

The datasets presented in this study can be found in online repositories. The names of the repository/repositories and accession number(s) can be found in the article/Supplementary material.

## Author contributions

FW designed and performed the research, analyzed transcriptomic data, developed EST-SSR primers, and wrote the manuscript. TT and TM collected the experimental samples. YD and XG participated in the genetic diversity analysis, participated in the revision of the manuscript. JY conceived this work, revised, and approved the final version of the manuscript. All authors contributed to the article and approved the submitted version.

## Funding

This work was supported by the Science and Technology Planning Project of Enshi Tujia and Miao Autonomous Prefecture (XYJ2022000027), the Science Funds for Young Scholar of Hubei Academy of Agricultural Sciences (2023NKYJJ30), Central government guides local science and technology development fund projects (2022BGE256), China Agriculture Research System (CARS-21), and the Forestry Science and Technology Project of Central Finance (2021TG16).

## Conflict of interest

The authors declare that the research was conducted in the absence of any commercial or financial relationships that could be construed as a potential conflict of interest.

## Publisher’s note

All claims expressed in this article are solely those of the authors and do not necessarily represent those of their affiliated organizations, or those of the publisher, the editors and the reviewers. Any product that may be evaluated in this article, or claim that may be made by its manufacturer, is not guaranteed or endorsed by the publisher.

## Footnotes

^1^ https://ngdc.cncb.ac.cn/gsub/

^2^ http://pgrc.ipk-gatersleben.de/misa/

^3^ http://taylor0.biology.ucla.edu/STRuctureHarvester/
