# Supplementary material for: Development of EST-SSR primers and genetic diversity analysis of the southern blight pathogen Sclerotium rolfsii using transcriptome data
Source: Front Microbiol. 2023 May 30;14:1152865. doi: 10.3389/fmicb.2023.1152865 (PMC10267981; doi:10.3389/fmicb.2023.1152865)
Supplement: Supplementary file 1 [file Data_Sheet_1.doc]

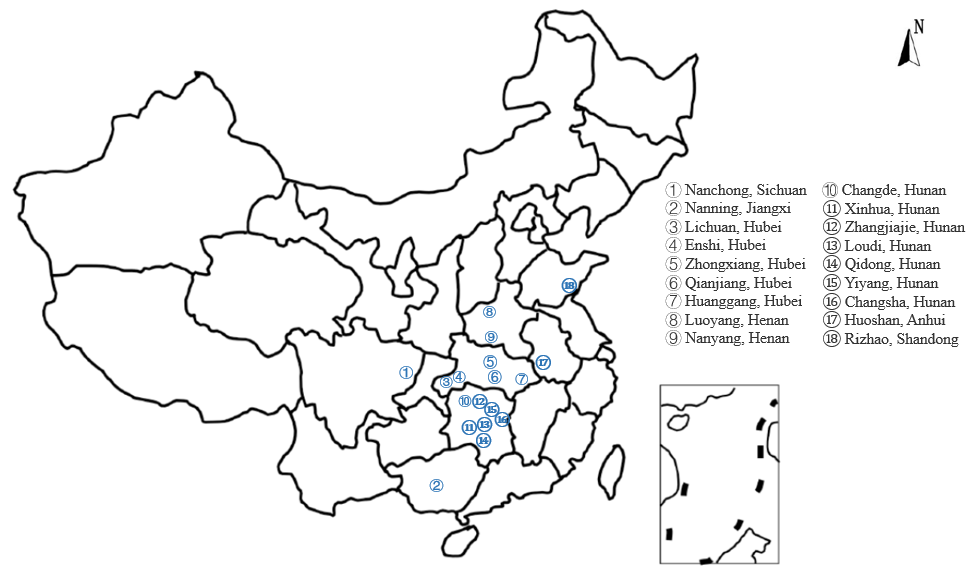


Figure S1. Sampling locations of Chinese southern blight.


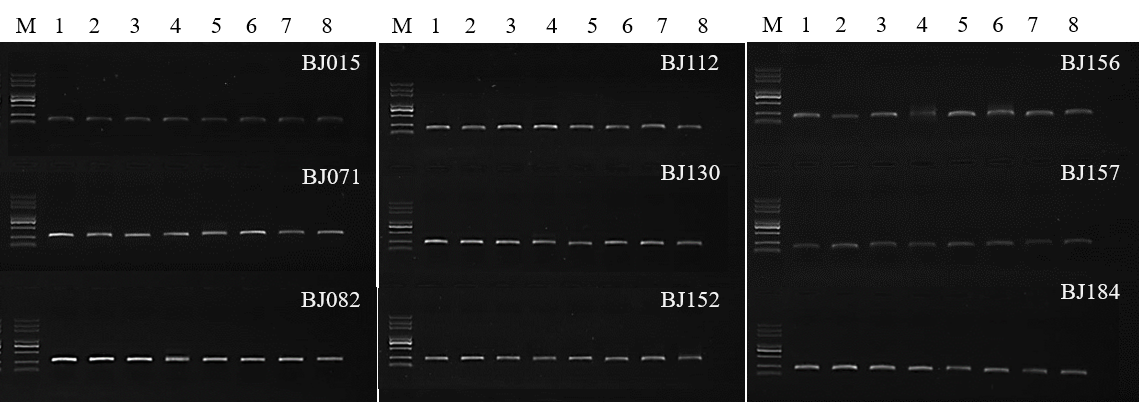


Figure S2. Agarose gel electrophoresis of 8 strains of *S. rolfsii* using the SSR primers

Lane M indicates the DL5000 DNA Marker; Lanes 1-8 indicate the 8 strains of *S. rolfsii*.


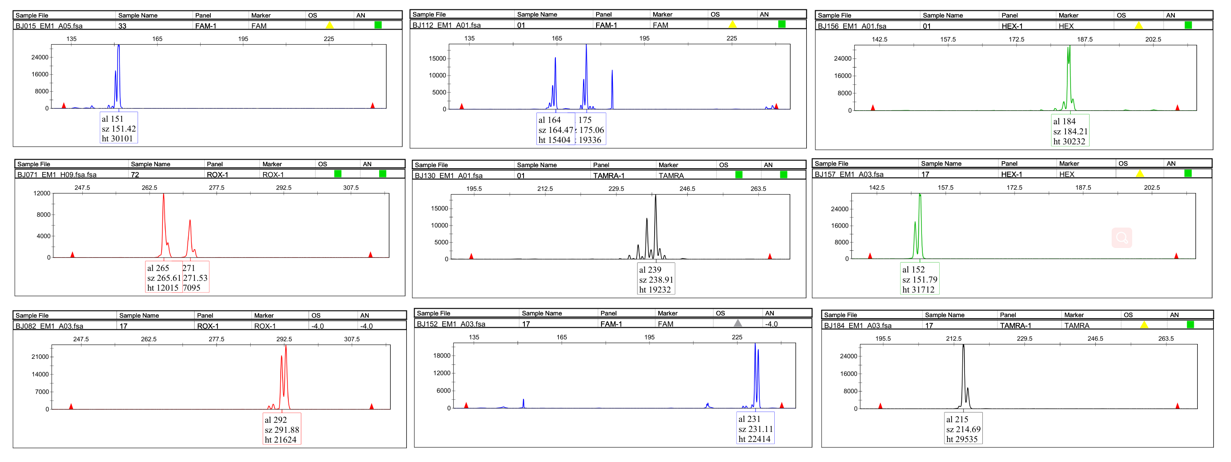


Figure S3. Capillary electrophoresis diagram of *S. rolfsii* amplification using SSR primers
